# Microwave-Assisted Kinetic Resolution of Homochiral (*Z*)-Cyclooct-5-ene-1,2-diol and (*Z*)*-*2-Acetoxycyclooct-4-enyl Acetate Using Lipases

**DOI:** 10.3390/molecules19079215

**Published:** 2014-07-02

**Authors:** Hervé Rouillard, Emmanuel Deau, Lisianne Domon, Jean-René Chérouvrier, Marianne Graber, Valérie Thiéry

**Affiliations:** Université de La Rochelle, UMR CNRS 7266 LIENSs, Avenue Crépeau, La Rochelle 17042, France; E-Mails: herve.rouillard@univ-lr.fr (H.R.); emmanuel.deau@univ-orleans.fr (E.D.); lisianne.domon@univ-lr.fr (L.D.); jcherouv@univ-lr.fr (J.-R.C.); marianne.graber@univ-lr.fr (M.G.)

**Keywords:** biocatalysis, microwave irradiation, lipase, homochiral diols, kinetic resolution

## Abstract

Over the last decade, the use of biocatalysts has become an attractive alternative to conventional chemical methods, especially for organic synthesis, due to their unusual properties. Among these enzymes, lipases are the most widely used, because they are cheap, easily available, cofactor-free, and have broad substrate specificity. Combined to microwave heating in non-aqueous medium, recent results suggest that irradiation may influence the enzyme activity. This Communication reports the benefits of lipases and the microwave irradiation on the kinetic resolution of racemic homochiral (*Z*)-cyclooct-5-ene-1,2-diol and (*Z*)*-*2-acetoxycyclooct-4-enyl acetate. In order to best achieve the kinetic resolution, different parameters were studied including the type of lipase, the temperature, the impact of microwave power compared to conventional heating. Optimization of the reaction parameters lead to the obtainment of highly enriched or enantiopure diols and diesters in a clean, efficient and safe way.

## 1. Introduction

Enantiomerically pure vicinal diols are versatile chemical scaffolds for the production of flavors and fragances. As part of our work on the enantioselective synthesis of methyl jasmonate derivatives from optically active bicyclo[3.3.0]octane derivatives by transannular cyclization, we first needed to prepare enantiopure homochiral (1*R*,2*R*)- and (1*S*,2*S*)-5-cyclooctene-1,2-diols. In recent years, the development of biocatalysts for organic synthesis has become an attractive alternative to conventional chemical methods. Among those biocatalysts, lipases have become very popular in both academic and industrial sectors because they are inexpensive, easily available, cofactor free and have a broad substrate specificity [1]. We decided to focus our interest on microwave-assisted lipase- mediated kinetic resolution involving CaLB (lipase B from *Candida antartica*) or PS (*Pseudomonas cepacia*)-catalyzed acetylation of diol. The use of microwave irradiation in biocatalysis can enhance the enzyme activity, for example in resolution reaction, in specific oxido-reduction reaction or hydrolysis. Combined to non-aqueous medium, recent results suggest that microwave irradiation can have also influence the enzyme stability and activity, in addition to altering/enhancing reaction rates and/or enantioselectivities, called non-thermal microwave effects [2,3,4,5,6,7,8]. However, the exact role of microwave irradiation on enzymes still remains unresolved [9]. To better comprehend the influence of microwave irradiation on a biocatalyst, we decided to compare the lipase-catalyzed resolution of difunctionalized compounds, under conventional and microwave irradiation heating. We report herein our studies on microwave assisted lipase resolution of homochiral (1*R*,2*R*)- and (1*S*,2*S*)-5-cyclooctene-1,2-diols **2** and their diesters **4**, by varying the irradiation power and reaction temperature.

## 2. Results and Discussion

**Scheme 1:**
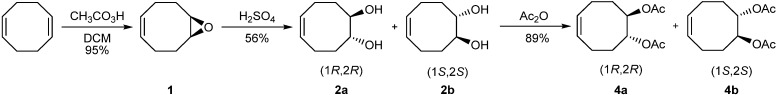
Epoxidation and hydrolysis of cycloocta-1,5-diene leading to racemic diols **2** and acetylation affording diacetates **4**.

In the context of the growing general interest for reducing energy costs, heating chemical reactions under microwave irradiation is a useful approach for achieving higher reaction kinetics and synthesizing cleaner products [10,11,12,13]. In combination with microwave technology and lipases, we wished to examine the synthesis of chiral cyclooctenic diols and diesters starting from cycloocta-1,5-diene using microwave technology. *rac*-Diols **2** and rac-diacetates **4** were initially prepared from cycloocta-1,5-diene in a three-steps sequence including epoxidation, ring opening with aqueous sulfuric acid, followed by acetylation with acetic anhydride (Scheme 1) [14,15].

The preparation of optically active 5-cyclooctene-1,2-diol **2** was first conceived by using microwave-assisted lipase-catalyzed desymmetrization of *meso*-symmetric diol **2** using vinyl acetate as the acylating agent in THF as solvent rather than isooctane or 2-methylbutan-2-ol, that gave lower results. In order to perform the reaction under microwave irradiation, we decided to choose thermostable immobilized lipases capable of withstanding microwave irradiation: Novozyme 435^®^ (CaLB immobilized on acrylic resin) and PS-D (*Pseudomonas cepacia* immobilized on diatomite). The goal was to obtain an enantioselective enhancement with immobilized lipases, as previous studies performed in our laboratory showed that resolution of *rac*-diol **2** with free *Pseudomonas cepacia* lipase at 55 °C in THF during 7 days afforded with a good conversion *rac*-monoacetate **3** (47%, 0% ee) and *rac*-diol **2** (51%, 0% ee) but with no selectivity at all. Indeed, by immobilization of enzymes onto solid supports, enhanced enzyme activity, selectivity, stability, and reusability in organic media may be achieved compared to the native enzyme [16].

**Scheme 2:**
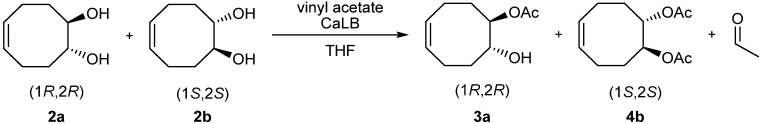
Enantioselective acetylation of diol (**2**) using immobilized CaLB lipase and vinyl acetate by classical heating at various temperatures.

Performed at 35 °C under conventional heating immobilized CaLB-enzymatic acylation of *rac*-diol (**2**) afforded after 3 weeks 28% of (1*R*,2*R*)-monoacetate **3a** (42% ee) and 6% of (1*S*,2*S*)-diacetate **4b** with an excellent 99% ee. A higher temperature (50 °C) led after 7 days to modest yields of monoacetate **3a** and a real enhancement of yield for diacetate **4b** (20% with ee > 99%) (Scheme 2 and Table 1).

**Table 1:** Enantioselective acetylation of diol (**2**) using immobilized CaLB lipase and vinyl acetate by classical heating at various temperatures.

Heating Mode	Temperature (°C)	Time	Monoacetate 3a Yield (%)	ee (%)	Diacetate 4b Yield (%)	ee (%)
Classical	35	3 weeks	28	42	6	>99
Classical	50	7 days	30	50	20	>99
Classical	50	14 h	traces		-	

**Scheme 3:**
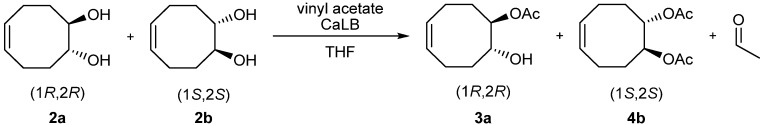
Enantioselective acetylation of diol **2** using immobilized CaLB with vinyl acetate lipase under microwave irradiation.

Under microwave irradiation at 35 °C (5 W), racemic diol **2** proceeded to give (1*R,*2*R*)-monoacetate **3a** (32%, 45% ee), trace amounts of (1*S*,2*S*)-diacetate **4b** (5%, >99% ee) and 65% of diol **2** (23% ee). In order to study the influence of the irradiation power on the biocatalytic media, we decided to apply a constant power (up to 300 W) while maintaining the temperature at 35 °C by using a microwave oven combined with a Coolmate^®^. We noticed an enhancement of the yield and the enantiomeric ratio of (1*R*,2*R*)-monoacetate **3a** (42%, 67% ee) and diol **2** (51%, 50% ee) and only 2% of diacetate **4b** (99% ee). At 50 °C (10W, 14 h) the same reaction yielded only esters: 58% of (1*R*,2*R*)-monoacetate **3a** with 55% ee and 37% of diacetate **4b** (99% ee). A higher temperature (80 °C, 40 W, 14 h) afforded a decrease of diacetate yield with a lower enantiomeric excess (30% of **4b** with 94% ee) (Scheme 3 and Table 2).

**Table 2:** Enantioselective acetylation of diol **2** using immobilized CaLB lipase under microwave irradiation in a large range of temperatures. * Constant power when the temperature is reached (max: 300 W, 2 min); ** Using Coolmate.

Power (W)	Incubation Time (h)	Temperature (°C)	Monoacetate 3a		Diaceate 4b	
			yield (%)	ee (%)	yield (%)	ee (%)
5 *	14	35	32	45	5	99
10 *	14	50	58	55	37	99
20 *	14	80	55	57	30	94
40 *	14	100	-	-	-	-
300 **	14	35	42	67	2	>99

These results suggest that, at higher temperature (100 °C), there is a loss of enzyme activity due to its denaturation. At 80 °C, Poojari *et al*., have shown that the immobilized-CalB (Novozym 435) was very stable and kept 90% of its activity after an incubation in diphenylether for 24 h [17]. The irradiation power appears to display a key role in enzyme properties, and best enhancement of monoacetate yield and ee was observed at 35 °C with 300 W. Brimble *et al*., have shown that compared to conventional heating the microwave irradiation led to higher conversion and enantiomeric excesses, in the case of lipase-catalyzed kinetic resolution of racemic secondary alcohols through acetylation [18].

**Scheme 4:**
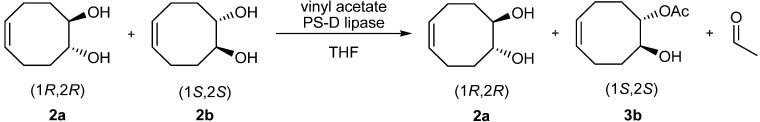
Acetylation of diol **2** using immobilized PS lipase (PS-D).

At 50 °C under conventional heating, enzyme-catalyzed acylation of rac-diol **2** with immobilized PS-D provided both (1*S*,2*S*)-monoacetate **3b** and (1*R*,2*R*)-diol **2a** in poor enantiomeric purity (respectively 45% ee for **3b** and 3% ee for **2a**) and poor conversion (6%). At higher temperatures (80, 100 °C), PS-D proved to be ineffective and lost all enzymatic activity. Under microwave irradiation at 50 °C (15 W), lipase resolution of racemic diol **2** afforded (1*S*,2*S*)-monoacetate **3b** (41%, 50% ee) and (1*R*,2*R*)-diol (57%, 35% ee). The microwave irradiation method gave a higher conversion compared to conventional heating. At 80 °C (35 W), the reaction yielded 12% of **3b** (35% ee) and 66% of diol with no selectivity (5% ee) (Scheme 4 and Table 3).

**Table 3:** Acetylation of diol **2** using immobilized PS lipase (PS-D) (incubation time:14 h). Effect of the heating mode and temperature on the conversion and kinetic resolution of rac-**2** into diol **2a** and monoacetate **3b**. * Constant power when the temperature is reached (max:300 W and 2 min).

Heating Mode	Temperature (°C)	Diol 2aee (%)	Monoacetate 3b yield (%)	ee (%)
Classical	50	3	6	45
	80	-	-	-
	100	-	-	-
Microwave	50 (15 W *)	35	41	50
	80 (35 W *)	5	12	35
	100 (closed vessel) (40 W *)	-	-	-

Noteworthy, PS-D exhibited a reverse enantiopreference for the monoacetate (*1S,2S*) compared to CaLB.

**Scheme 5:**
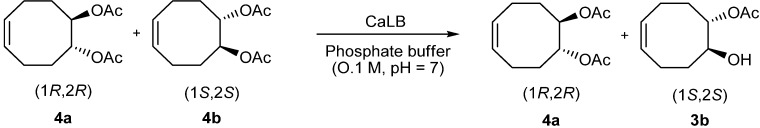
Hydrolysis of diacetate **4** using immobilized CaLB lipase in phosphate buffer (0.1 M, pH 7). For detailed conversion and ee, see Table 4.

Following these preliminary results, we then investigated the lipase-catalyzed kinetic hydrolytic resolution of *rac*-diacetate **4** according to the Suemune procedure [19]. Instead of using PFL (*Pseudomonas fluorescens* lipase), we used immobilized CaLB and PS-D in order to perform later the reaction under microwave. Performed at various temperature (35, 50 and 80 °C) under conventional heating CaLB-catalyzed hydrolysis of *rac*-diacetate (**4**) in phosphate buffer (0.1 M, pH 7.0) led only to traces of monoacetate at 50 °C. Compared to conventional heating, the microwave irradiation at 50 °C during 14 h led to a higher conversion (20%) with excellent enantiomeric excess for monoacetate (ee: 97% for **3b** (1*S*,2*S*)) besides diacetate **4a** (ee: 34%) (Scheme 5 and Table 4).

**Table 4:** Hydrolysis of *rac*-diacetate **4** using immobilized CaLB lipase (incubation time: 14 h). Effect of the heating mode and temperature on the conversion and kinetic resolution of rac-**4** into diacetate **4a** and monoacetate **3b**. * Constant power when the temperature is reached (max: 300 W and 2 min).

Heating Mode	Temp. (°C)	Conversion	Diacetate 4a ee (%)	Monoacetate 3b ee (%)
Classical	35	-	-	*-*
	50	Traces	-	*-*
	80	-	-	*-*
Microwave	35 (1–5 W *)	-	-	*-*
	50 (5 W *)	20%	34	97
			[α]_D_ = +29°	[α]_D_ = +6°
	80 (10 W *)	-	-	*-*

**Scheme 6:**
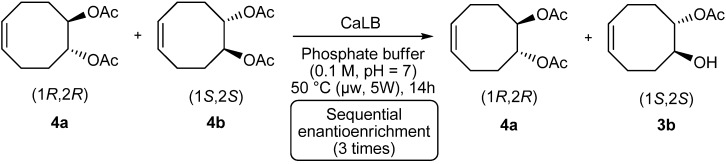
Sequential enantioenrichment of diacetate **4a**.

Given the promising results obtained during the first enantioenrichment of *rac*-**4** with CaLB, the recovered (1*R,*2*R*)-diacetate **4a** with 34% ee was submitted twice to the same enzymatic hydrolysis under microwave irradiation (2 × 14 h, 50 °C, 5 W). Enantiopure (1*R*,2*R*)-diacetate **4a** was obtained in 49% yield from *rac*-**4** (Scheme 6 and Table 5).

**Table 5:** Sequential enantioenrichment of diacetate **4a**.

	Yield (%)	Monoacetate 3b ee (%)	[α]_D_	yield (%)	Diacetate 4a ee (%)	[α]_D_
1st enrichment	20	97	+5.8°	80	34	+25°
2nd enrichment	41	98	+6°	57	72	+57°
3rd enrichment	51	97	+5.7°	49	>99	+81°

**Scheme 7:**
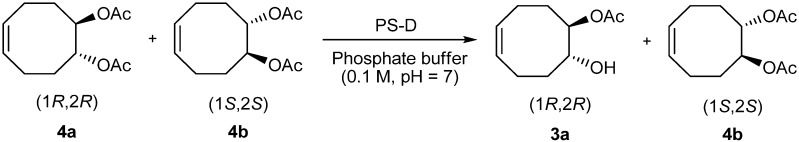
Hydrolysis of diacetate 4 using PS-D lipase in phosphate buffer (0.1 M, pH 7).

CaLB and the PS-D were finally compared for their ability to carry out an effective kinetic resolution of rac-**4** diester. The hydrolysis using PS-D was conducted in the same manner. In classical conditions whatever the temperature (35, 50, 80 °C) and the reaction time (14 h, 7 days), in no case conversion to monoacetate **3** was observed. Interestingly, under microwave irradiation at 50 °C (14 h), *rac*-**4** was desymmetrized into (1*R*,2*R*) monoacetate **3a** with 81% ee and (1*S*,2*S*)-diacetate **4b** (28% ee), highlighting a non-thermal effect (Scheme 7 and Table 6).

The microwave-assisted hydrolysis seems to enhance the enzyme activity compared to classical heating. Comparing the results with Suemune *et al*. [19], the CaLB seems to be a convenient enzyme in microwave irradiation resulting in the obtention of enantiopure monoacetate **3a** in a quick and clean way. However, the same reaction with PS-D needed to be optimized to obtain a better ee.

**Table 6:** Hydrolysis of diacetate **4** using PS-D lipase (incubation time: 14 h). Effect of the heating mode and temperature on the conversion and kinetic resolution of rac-**4** into monoaceate **3a** and diester **4b**. * Constant power when the temperature is reached (max: 300 W and 2 min).

Mode of Heating	Temp. (°C)	Conversion	Monoacetate 3a ee (%)	Diacetate 4b ee (%)
Classical	35	-	*-*	-
	50	-	*-*	-
	80	-	*-*	-
Microwave	35 (1–5 W *)	-	*-*	-
	50 (5 W *)	10%	81	28
			[α]_D_ = −4°	[α]_D_ = −24°
	80 (10 W *)	-	*-*	-

**Scheme 8:**
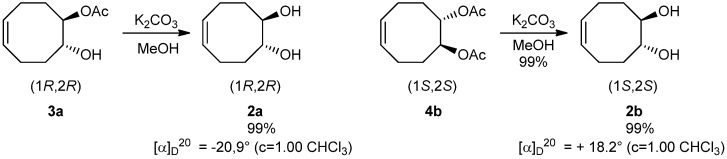
Isolation of enantiopure diols **2a** and **2b** by methanolysis of monoacetate **3a** and diacetate **4b**.

Finally, the isolated enantiopure monoacetates **3a**, **3b** and diacetates **4a** and **4b** were then quantitatively converted into (1*R*,2*R*)-diol **2a** and (1*S*,2*S*)-diol **2b** with >99% ee by methanolysis [7] (Scheme 8).

## 3. Experimental Section

### 3.1. General Information

Lipases from *Pseudomonas cepacia* (immobilized on diatoms MKBB3465, 500 PLU^−1^) and *Candida antarctica* (immobilized on acrylic resin 077K1155, 10,000 PLU^−1^ *Novozym 435*^®^ or free form) were purchased from Sigma Aldrich (Sigma-Aldrich Chemie S.a.r.l., Saint Quentin Fallavier, France). All other chemicals were purchased from Sigma Aldrich and were used without further purification except in the case of vinyl acetate which was used after fresh distillation.

IR spectra were recorded on a Perkin–Elmer Spectrum 100 IRFT-ATR instrument (Perkin-Elmer, Courtaboeuf-Les Ulis, France). ^1^H- and ^13^C-NMR were recorded on a JEOL JNM LA400 (400 MHz) spectrometer (JEOL SAS Europe, Croissy Sur Seine, France). Chemical shifts (δ) are reported in parts per million (ppm) downfield from tetramethylsilane (TMS) which was used as internal standard. Coupling constants J are given in Hz. The high resolution mass spectra (HRMS) were recorded on a Varian MAT311 spectrometer (Agilent Technologies SAS, Les Ulis, France) in the Centre Régional de Mesures Physiques de l’Ouest (CRMPO), Université de Rennes. Analytical thin layer chromatography (TLC) was performed on Merck Kieselgel 60 F254 aluminum packed plates (Merck KgaA, Darmstadt, Germany).

Enantiomeric ratios were determined by gas chromatography (Agilent 7890A, Agilent Technologies SAS, Les Ulis, France) equipped with an autosampler (7688B) and flame ionization detector (FID). For the experiment, a CP-Chirasil-Dex (0.25 mm × 25 m × 0.25 µm, Chromopack, Agilent Technologies SAS, Les Ulis, France) column was used. The injector and the detector were kept at 180 °C. Nitrogen was used as gas carrier at a flow of 1.5 mL/min. Hydrogen, air and nitrogen were supplied to the FID at 35 mL·min^−1^, 350 mL·min^−1^ and 25 mL·min^−1^ respectively. The products are analyzed at 110 °C. The enantiomeric ratio and yields were calculated by taking the average of two duplicates, with an error <2%.

High performance liquid chromatography (HPLC) was carried out in a Waters 600 s system (Water SAS, Guyancourt, France) combined with an autosampler (Waters 717 plus). A Chiralpak–AD column (amylose tris-(3,5-dimethylphenylcarbamate) coated on 10 μm silica-gel, Daicel Chemical, 250 × 4.6 mm, Chiral Technologies Europe SAS, Illkirch, France) is used. Eluent was *n*-heptane/EtOH, 9/1 at a flowrate of 1 mL·min^−1^. Products were analyzed using a differential refractometer (Waters 410). Optical rotations were measured on a Perkin Elmer 341 polarimeter.

Microwave reactions were conducted using a CEM Discover^®^, single mode operating system (CEM France, Saclay, France) working at 2.45 GHz, with a programmable power ranging from 1 to 300 W. The microwave can be equipped with a Coolmate^®^ system allowing reactions with high power input (up to 300 W) while maintaining the reaction media at 35 °C. This system is cooled by a cryogenic fluid (Galden HT-55^®^, BT Electronics, Les Ulis, France). In closed vessel mode, microwave irradiation experiments were carried out using a single-mode microwave instrument (Initiator, Biotage, Uppsala, Sweden) working at 2.45 GHz, with a power programmable from 1 to 450 W (0–20 bars).

### 3.2. (Z)-(1S,8R)-9-Oxa-Bicyclo[6.1.0]Non-4-Ene (**1**)

Under inert atmosphere, to a solution of cycloocta-1,5-diene (20 g, 0.161 mol) and sodium carbonate (117.6 g, 1.11 mol) in dichloromethane (560 mL) stirred at 0 °C was added dropwise a solution of peracetic acid (42.7 mL, 0.222 mol) in dichloromethane (500 mL) during 2 h. The mixture was stirred for 8 h at 0 °C and 12 h at room temperature. The mixture was quenched with 500 mL of water and extracted with (3 × 250 mL) of dichloromethane. The organic layers were dried with magnesium sulfate anhydrous and concentrated under reduced pressure. The crude residue was purified by column chromatography (silica gel, petroleum ether/ethyl acetate 90/10) to provide the (*Z*)-(1*S,*8*R*)-9-oxa-bicyclo[6.1.0]non-4-ene (**1**, 5.697 g, 67% yield) as a colorless oil; ν_max_ (cm^−1^): 3003, 2906, 2887, 1655, 1445, 1428, 1228, 1669, 1099, 1039, 934, 762, 743; ^1^H-NMR (CDCl_3_): δ_H_ 5.56–5.60 (2H, m, CH = CH), 3.00–3.02 (2H, m, CHCH), 2.41–2.47 (2H, m, CH_2_), 2.08–2.15 (2H, m, CH_2_), 1.98–2.17 (4H, m, 2×CH_2_); ^13^C-NMR (CDCl_3_): δ_C_ 128.6, 56.2, 27.8, 23.4.

### 3.3. (Z)-Cyclooct-5-en-1,2-diol (**2**)

Under inert atmosphere, to the (*Z*)-(1*S,*8*R*)*-*9-oxabicyclo[6.1.0]non-4-ene (**1**, 5.697 g, 45.9 mmol) vigorously stirred was added a solution of sulfuric acid 2 M (25.3 mL, 50.47 mmol). After 4 h under stirring, the mixture was extracted with 3 × 75 mL of ethyl acetate. The organic layers were washed with a saturated solution of sodium hydrogenocarbonate (40 mL), brine (40 mL), dried with magnesium sulfate and concentrated under vacuum. The crude residue was purified by column chromatography (silica gel, petroleum ether/ethyl acetate 60/40) to give the *rac*-(*Z*)-cyclooct-5-en-1,2-diol (**2**, 3.691 g, 57% yield) as a colorless oil; ν_max_ (cm^−1^): 3362, 3014, 2964, 2861, 1651, 1427, 1429, 1400, 1271, 1202, 1010, 994, 976, 947, 868, 732, 719; ^1^H-NMR (CDCl_3_): δ_H_ 5.55–5.59 (2H, m, CH = CH), 3.57–3.61 (4H, m, CHOHCHOH), 2.29–2.35 (2H, m, CH_2_), 2.00–2.12 (4H, m, 2×CH_2_), 1.52–1.58 (2H, m, CH_2_); ^13^C-NMR (CDCl_3_): δ_C_ 128.9, 73.8, 33.1, 22.6; HRMS: calculated for C_8_H_14_O_2_ [M]^+^: 142.09938, Found: 142.1001 (5 ppm).

### 3.4. (1R,2R)-(Z)-1-Hydroxycyclooct-4-enyl Acetate (**3a**)

Compound **3a** was obtained as a colorless oil 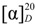 −3.0° (*c* 1.00 CHCl_3_). ν_max_ (cm^−1^): 3449, 3012, 2936, 1717, 1654, 1430, 1235, 1030, 971, 933, 720; ^1^H-NMR (CDCl_3_): δ_H_ 5.59–5.69 (2H, m, CH = CH), 4.95 (1H dt, *J* = 8.8 Hz, 4.0Hz, CHOAc), 3.90 (1H, dt, *J* = 2.1 Hz, CHOH), 2.54 (1H, s, OH), 2.38–2.42 (m, 2H, CH_2_), 2.04–2.25 (m, 7H, 2×CH_2_ and CH_3_), 1.68–1.75 (m, 2H, CH_2_); ^13^C-NMR (CDCl_3_): δ_C_ 170.9, 129.6, 128.6, 77.3, 72.1, 32.8, 30.0, 22.8, 22.8, 21.2. GC: Cromopack column t_R_ = 59.7 min HRMS: calcd for C_8_H_14_O_2_ [M-CH_2_CO]^+^: 142.09938, Found: 142.0993 (0 ppm).

### 3.5. (1S,2S)-(Z)-1-Hydroxycyclooct-4-enyl Acetate (**3b**)

Compound **3b** was obtained as a colorless oil. 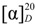 +3.2° (*c* 1.00 CHCl_3_). ν_max_ (cm^−1^): 3449, 3012, 2936, 1717, 1654, 1430, 1235, 1030, 971, 933, 720; ^1^H-NMR (CDCl_3_): δ_H_ 5.59–5.69 (2H, m, CH = CH), 4.95 (1H dt, *J* = 8.8 Hz, 4.0Hz, CHOAc), 3.90 (1H, dt, *J* = 2.1 Hz, CHOH), 2.54 (1H, s, OH), 2.38–2.42 (m, 2H, CH_2_), 2.04–2.25 (m, 7H, 2×CH_2_ and CH_3_), 1.68–1.75 (m, 2H, CH_2_); ^13^C-NMR (CDCl_3_): δ_C_ 170.9, 129.6, 128.6, 77.3, 72.1, 32.8, 30.0, 22.8, 22.8, 21.2. GC: Cromopack column t_R_ = 56.6 min HRMS: calcd for C_8_H_14_O_2_ [M-CH_2_CO]^+^: 142.09938, Found: 142.0993 (0 ppm).

### 3.6. (1R,2R)-(Z)-2-Acetoxy-cyclooct-4-enyle Acetate (**4a**)

Compound **4a** was obtained as a colorless oil. 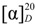 +81.0° (*c* 1.00 CHCl_3_). ν_max_ (cm^−1^): 3016, 2938, 2866, 1732, 1654, 1431, 1370, 1226, 1244, 1032, 978, 946, 735, 722; ^1^H-NMR (CDCl_3_): δ_H_ 5.69–5.62 (2H, m, CH = CH), 5.05–5.08 (2H, m, 2×CHOH), 2.35–2.41 (2H, m, CH_2_), 2.12–2.18 (2H, m, CH_2_), 2.01–2.04 (2H, m, CH_2_), 1.96 (s, 6H, 2×CH_3_), 1.73–1.76 (2H, m, CH_2_). ^13^C-NMR (CDCl_3_): δ_C_ 170.1, 128.6, 73.6, 29.9, 22.7, 20.9; [α]_D_: +83.9° (c = 1.00 CHCl_3_) GC Cromopack column t_R_ = 61.2 min.

### 3.7. (1S,2S)-(Z)-2-Acetoxy-cyclooct-4-enyle acetate (**4b**)

Compound **4b** was obtained as a colorless oil; 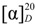 −79.0° (*c* 1.00 CHCl_3_). ν_max_ (cm^−1^): 3016, 2938, 2866, 1732, 1654, 1431, 1370, 1226, 1244, 1032, 978, 946, 735, 722; ^1^H-NMR (CDCl_3_): δ_H_ 5.69–5.62 (2H, m, CH = CH), 5.05–5.08 (2H, m, 2×CHOH), 2.35–2.41 (2H, m, CH_2_), 2.12–2.18 (2H, m, CH_2_), 2.01–2.04 (2H, m, CH_2_), 1.96 (s, 6H, 2×CH_3_), 1.73–1.76 (2H, m, CH_2_). ^13^C-NMR (DCl_3_): δ_C_ 170.1, 128.6, 73.6, 29.9, 22.7, 20.9; GC: Cromapack column t_R_ = 59.4 min; HRMS calcd for C_10_H_16_O_3_ [M-CH_2_CO]^+^: 184.10994, Found: 184.1091 (4 ppm).

### 3.8. General Procedure for Enantioselective Acetylation of Racemic (Z)–Cyclooct-5-en-1,2-diol (**2**) Using Lipase

Under inert atmosphere, to a solution of racemic diol **2** (0.2 g, 1.41 mmol) solubilized in 2.5 mL of THF were added the vinyl acetate (1.3 mL, 14 mmol) and the appropriate immobilized lipase (50 mg). The mixture was stirred at the required temperature under classical heating or microwave irradiation (see Table 1, Table 2 and Table 3). The mixture was filtrated, extracted with ethyl acetate (3 × 7 mL). The organic layers were washed with 3 mL of HCl 5%, 3 mL of sodium hydrogenocarbonate, brine, dried with magnesium sulfate and concentrated under reduced pressure. The crude residue was purified by column chromatography (silica gel, petroleum ether/ethyl acetate 60/40) to give the (*1R*,*2R*)-(*Z*)*-*1-hydroxy-cyclooct-4-enyl acetates **3a**, **3b**, **4b**.

### 3.9. General Procedure for the Hydrolysis of (Z)-2-Acetoxy-cyclooct-4-enyle Acetate (**4**) by Candida Antarctica Lipase B

Under inert atmosphere, to a solution of racemic (*Z*)*-*2-acetoxycyclooct-4-enyl acetate **4** (0.2 g, 0.88 mmol) in 2.5 mL of phosphate buffer 0.1 M, pH = 7.0, was added the lipase (50 mg). The mixture was stirred at 50 °C under microwave irradiation (see Table 4, Table 5 and Table 6). The mixture was filtrated, extracted with ethyl acetate (3 × 7 mL). The organic layers were washed with 3 mL of HCl 5%, 3 mL of sodium hydrogenocarbonate, brine, dried with magnesium sulfate and concentrated under reduced pressure. The crude residue was purified by column chromatography (silica gel, petroleum ether/ethyl acetate 60/40) to give **3a**, **3b** and **4a**, **4b**.

### 3.10. Diacetate Enrichment

The enriched (*Z*)*-*2-acetoxycyclooct-4-enyl compound **4a** is solubilized in 2.5 mL of phosphate buffer 0.1 M, pH = 7.0. *Candida antarctica* lipase B (50 mg) was added and the mixture was stirred at 50 °C during 14 h by microwave irradiation (open vessel). The mixture was filtrated, extracted with ethyl acetate (3 × 7 mL), The combined organic layers were washed with 3 mL of HCl 5%, 3 mL of sodium hydrogenocarbonate, brine, dried with magnesium sulfate and concentrated under reduced pressure. The crude residue was purified by chromatography column (silica gel, petroleum ether/ethyl acetate 60/40) to give the (*Z*)-(1*S*,2*S*)*-*2-hydroxy-cyclooct-4-enyl acetate **3b** in 49% overall yield (0.098 g, ee > 99%) and **4a** in 51% overall yield (0.083 g, ee > 99%).

### 3.11. Influence of the Power of the Microwave for the Acetylation of (Z)–Cyclooct-5-en-1,2-diol (**2**)

The cryogenic fluid (Garlon 80^®^) of the Coolmate^®^ was cooled by dry ice, and maintained at 7 °C. To a solution of racemic diol **2** (0.2 g, 1.406 mmol) solubilized in 2.5 mL of THF was added vinyl acetate (1.3 mL, 14 mmol) and *Candida antarctica* lipase (50 mg). The mixture was irradiated with an internal temperature set to 35 °C, leading to an irradiation power of 300 W. After 7 h of irradiation, the mixture was filtrated, extracted with ethyl acetate (3 × 7 mL). The organic layers were washed with 3 mL of HCl 5%, 3 mL of sodium hydrogenocarbonate, brine, dried with magnesium sulfate and concentrated under reduced pressure. The crude residue was purified by column chromatography (silica gel, petroleum ether/ethyl acetate 60/40) to give the (*Z*)*-*(1*R,*2*R*)-1-hydroxycyclooct-4-enyl acetate **3a** in 42% yield (0.108 g, ee = 67%), (*Z*)-(1*S,*2*S*)*-*2-acetoxycyclooct-4-enyl acetate **4b** in 2% yield (ee = 99%) and (Z)–cyclooct-5-en-1,2-diol in 51% yield (0.102 g, ee = 50%).

### 3.12. Synthesis of Diols by Saponification of Esters

Under inert atmosphere, to a solution of enantiopure (*Z*)-(1*R*,2*R*)*-*2-acetoxycyclooct-4-enyl acetate **4b** (0.2 g, 17.3 mmol) or enantiopure monoacetate **3a** (0.2 g, 12.0 mmol) in methanol (10 mL) was added potassium carbonate anhydrous (4 mg, 0.86 mmol). The mixture was stirred 8 h at 0 °C, and 10 mL of hydrochloric acid 1 M are added. The aqueous layer was extracted with ethyl acetate (3 × 8 mL), washed with a saturated solution of sodium hydrogenocarbonate (5 mL) and brine (5 mL). The organic layers were dried with magnesium sulfate, concentrated under reduced pressure.

### 3.13. (1R,2R)-(Z)-Cyclooct-5-ene-1,2-diol (**2a**)

The crude residue was purified by column chromatography (silica gel, petroleum ether/ethyl acetate 60/40) to give the (*Z*)–(1*R,*2*R*)-cyclooct-5-ene-1,2-diol **2a** (0.152 g, 99% yield) as a white solid; 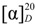 −20.9° (*c* 1,00 CHCl_3_); ν_max_ (cm^−1^): 3362, 3014, 2964, 2861, 1651, 1427, 1429, 1400, 1271, 1202, 1010, 994, 976, 947, 868, 732, 719; ^1^H-NMR (CDCl_3_): δ_H_ 5.55–5.59 (2H, m, CH = CH), 3.57–3.61 (m, 4H, 2×CHOH), 2.29–2.35 (2H, m, CH_2_), 2.00–2.12 (2H, m, CH_2_), 1.52–1.58 (2H, m, CH_2_); ^13^C-NMR (CDCl_3_): δ_c_ 128.9, 73.8, 33.1, 22.6; HRMS: calculated for C_8_H_14_O_2_ [M]^+^: 142.09938, Found: 142.1001 (5 ppm). HPLC t_R_ = 11.9 min.

### 3.14. (1S,2S)-(Z)-Cyclooct-5-ene-1,2-diol (**2b**)

The crude residue was purified by column chromatography (silica gel, petroleum ether/ethyl acetate 60/40) to give the (*Z*)–(1*S*,2*S*)-cyclooct-5-en-1,2-diol **2b** (0.153 g, 99% yield) as a white solid. 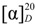 +18.2° (c = 1,00 CHCl_3_)ν_max_ (cm^−1^): 3362, 3014, 2964, 2861, 1651, 1427, 1429, 1400, 1271, 1202, 1010, 994, 976, 947, 868, 732, 719; ^1^H-NMR (CDCl_3_): δ_H_ 5.55–5.59 (2H, m, CH = CH), 3.57–3.61 (m, 4H, 2×CHOH), 2.29–2.35 (2H, m, CH_2_), 2.00–2.12 (2H, m, CH_2_), 1.52–1.58 (2H, m, CH_2_); ^13^C-NMR (CDCl_3_): δ_c_ 128.9, 73.8, 33.1, 22.6; HRMS: calculated for C_8_H_14_O_2_ [M]^+^: 142.09938, Found: 142.1001 (5 ppm). HPLC t_R_ = 10.8 min.

## 4. Conclusions

The kinetic resolution of homochiral (*Z*)-cyclooct-5-ene-1,2-diols and (*Z*)*-*2-acetoxycyclooct-4-enyl acetates has been accomplished controlled using two different lipases (CaLB or PS-D) in order to obtain one or the other useful enantiomer. The role of the microwave power has also been highlighted. Finally, by microwave irradiation, this eco-efficient optimization for the resolution of racemic diols, leads to a reduction of the reaction time and a decrease of power consumption, without any toxicity.

## Supplementary Materials

Supplementary materials can be accessed at: http://www.mdpi.com/1420-3049/19/7/9230/s1.
