# Supplementary material for: Microwave-Assisted Kinetic Resolution of Homochiral (Z)-Cyclooct-5-ene-1,2-diol and (Z)-2-Acetoxycyclooct-4-enyl Acetate Using Lipases
Source: Molecules. 2014 Jul 2;19(7):9215–27. doi: 10.3390/molecules19079215 (PMC6270989; doi:10.3390/molecules19079215)

Supplementary Materials

(1) Gas Chromatography

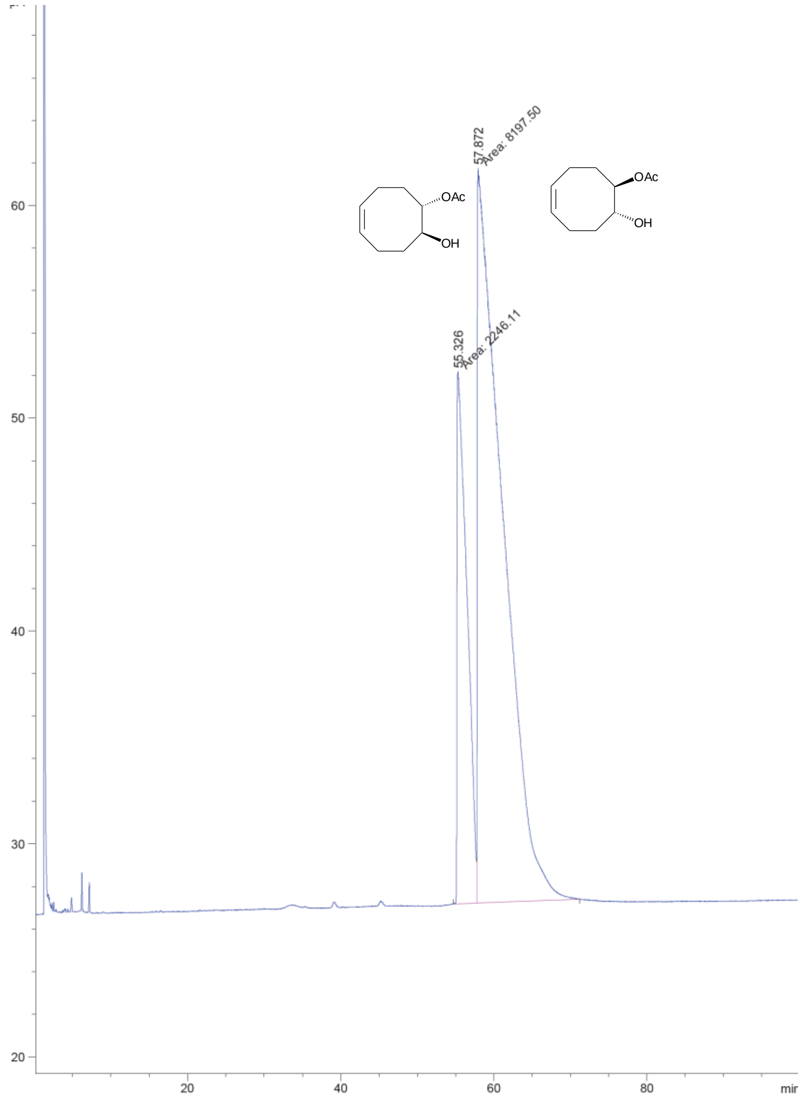

*Exemple of Monoacetate **3a** (ee=56%) obtained by acetylation using immobilized CALB lipase*

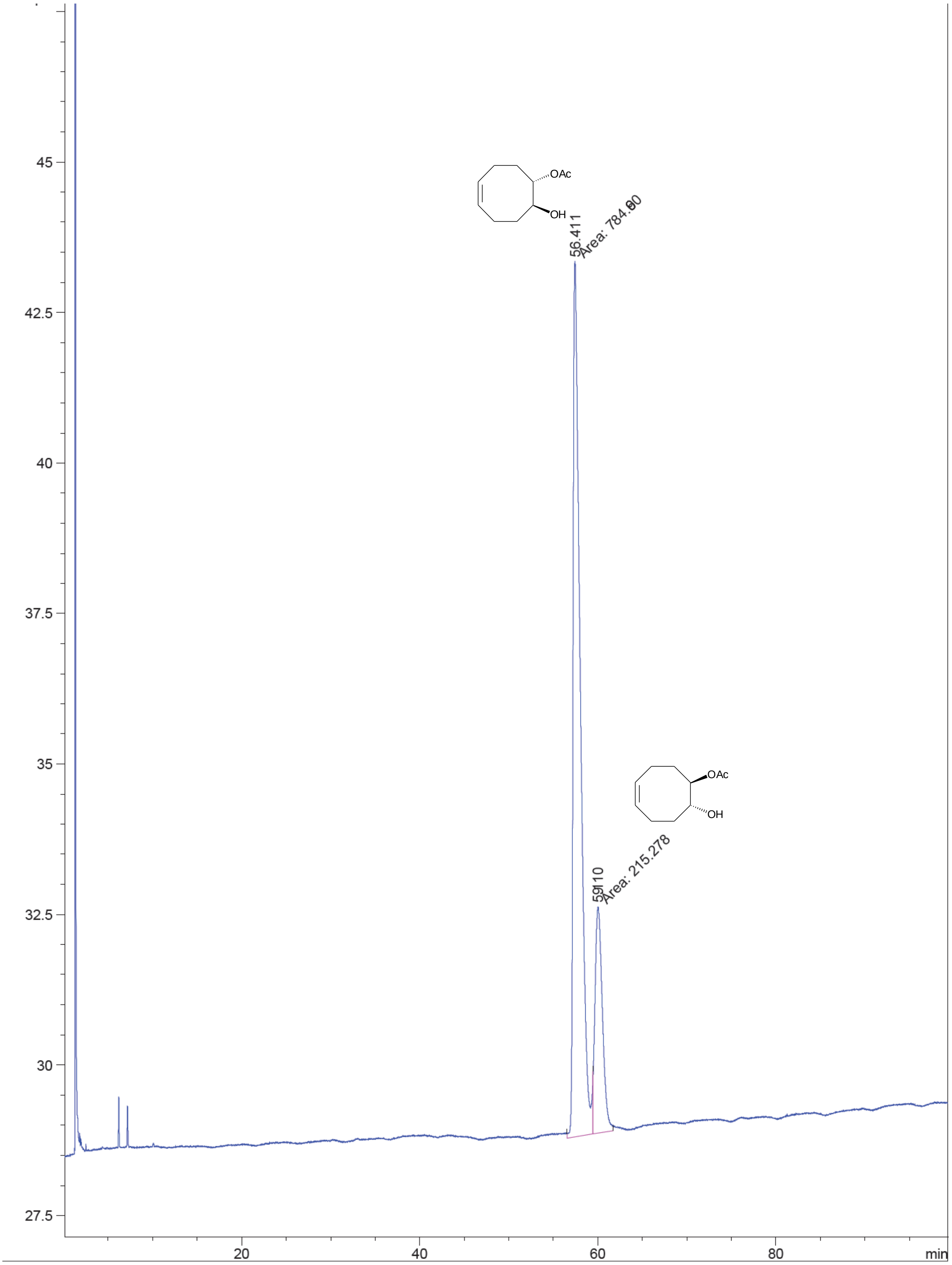

*Exemple of monoacetate **3b** (ee = 57%) obtained by acetylation using Immobilized Pseudomonas cepacia lipase*

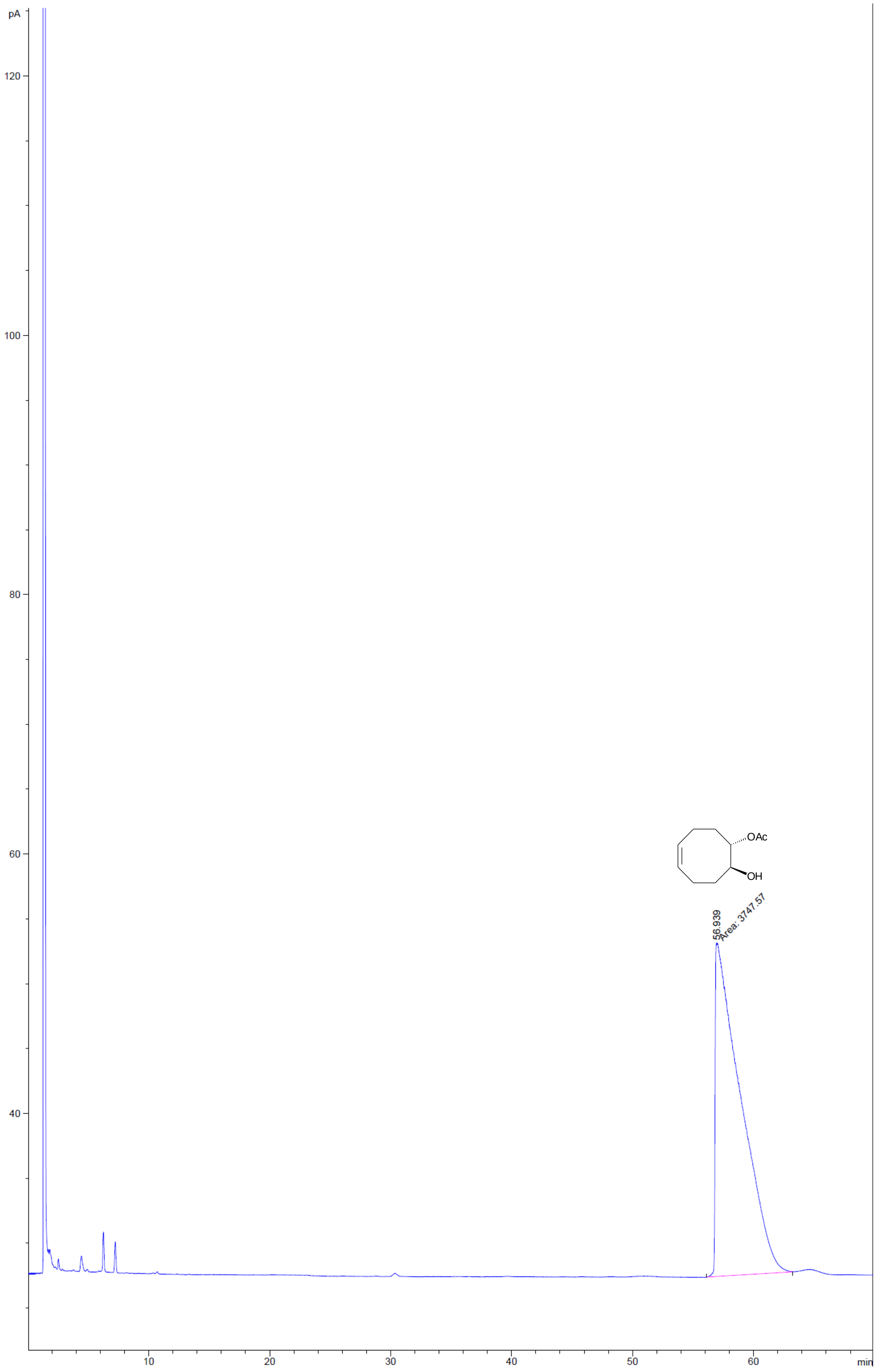

Enantiopure monoacetate ***3b***

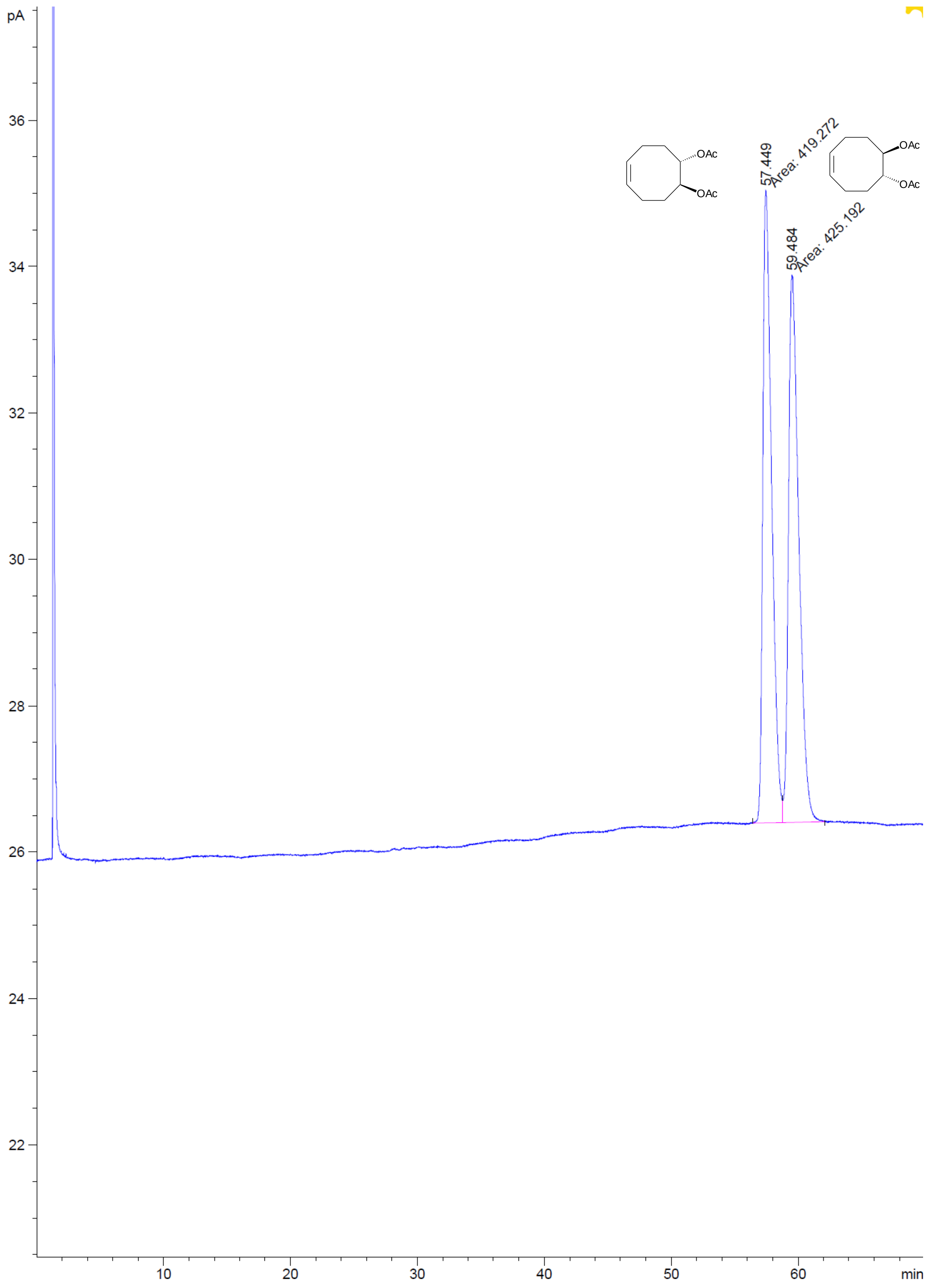

*Racemic diacetate **4***

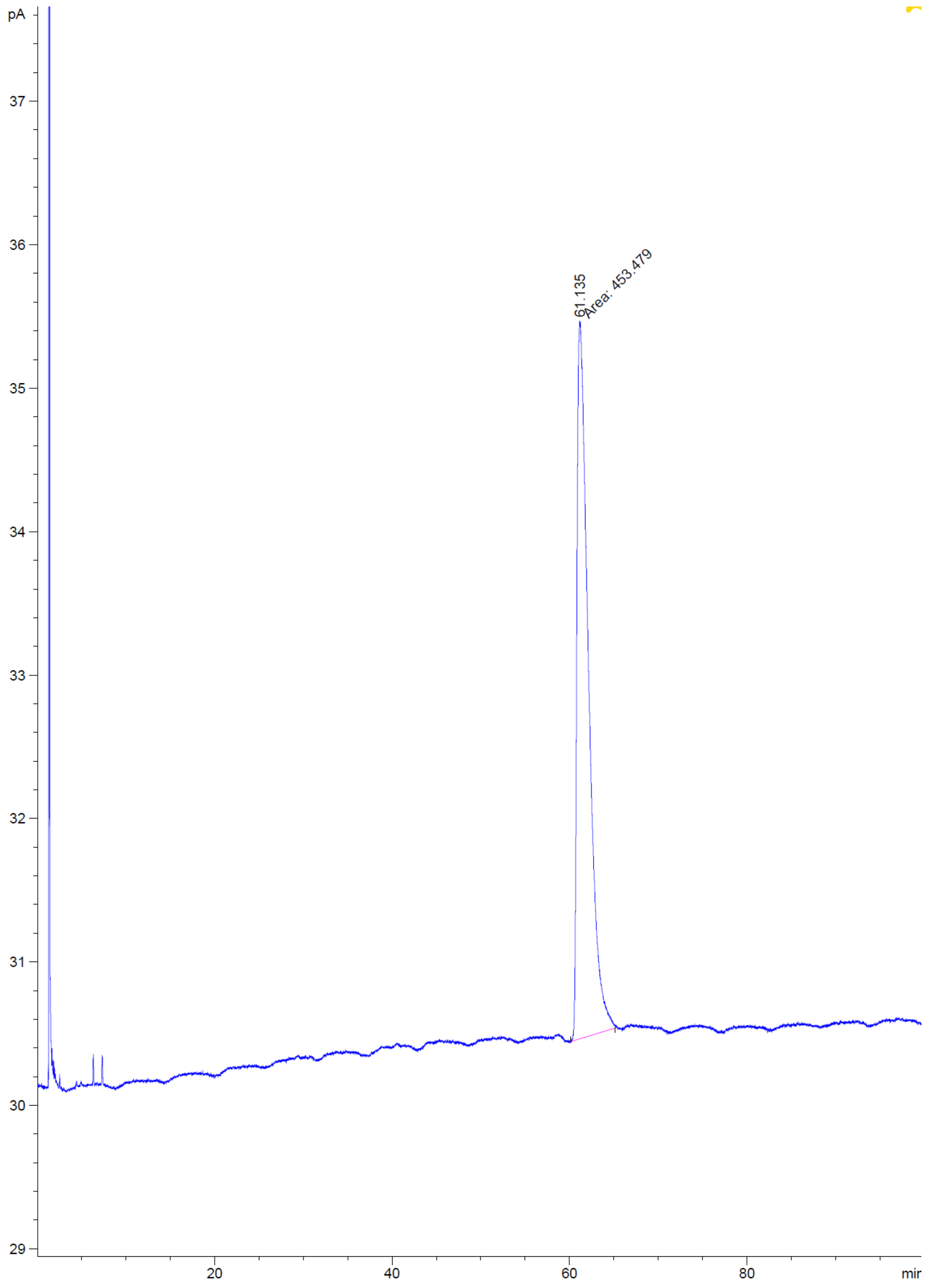

*Diacetate **4a** enriched*

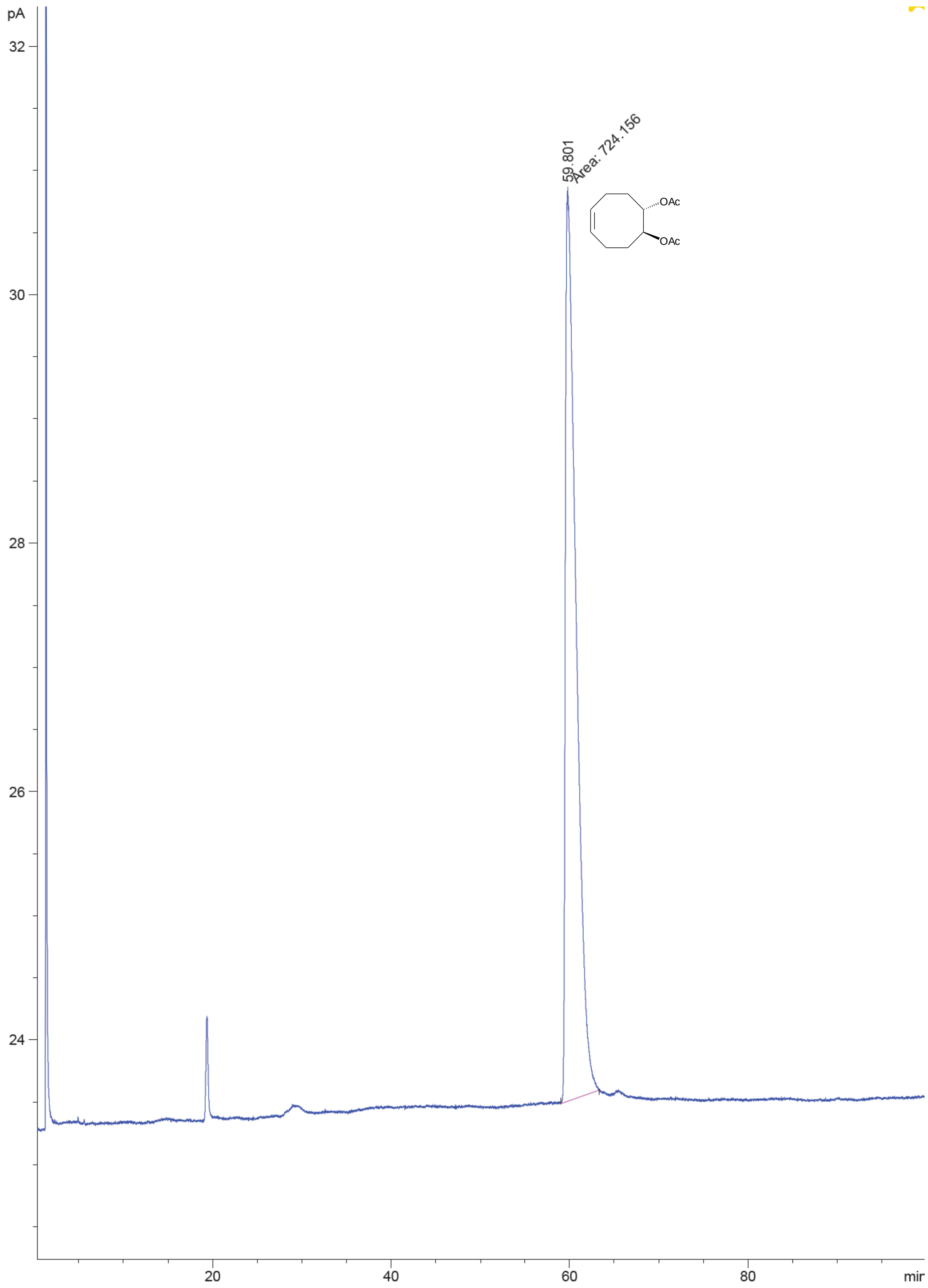

Diacetate ***4b***

2) HPLC

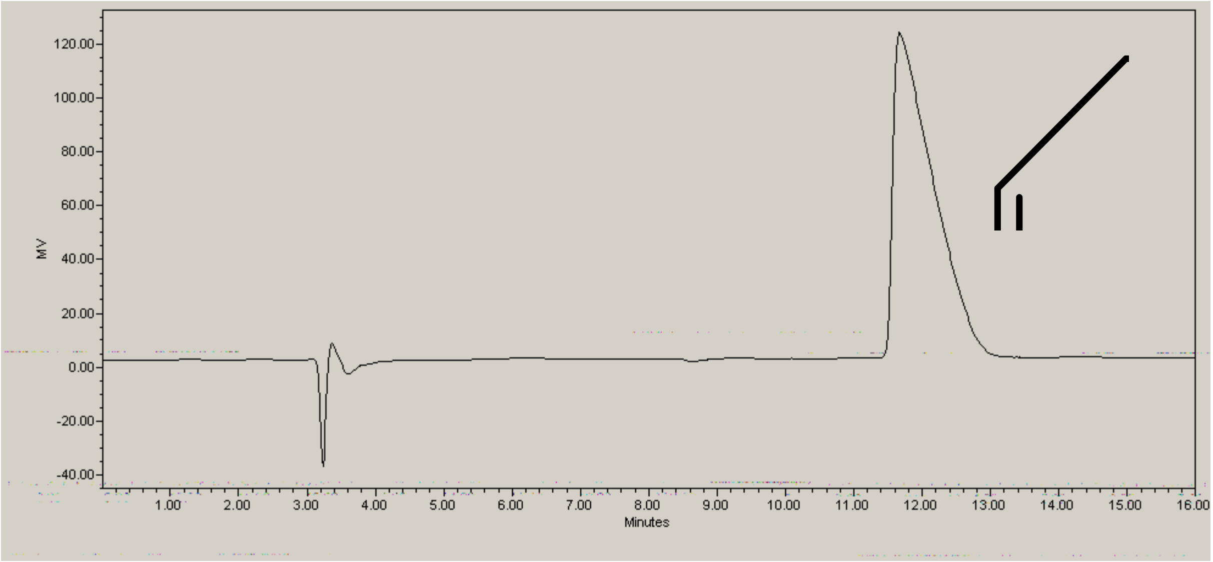

*Diol **2a***

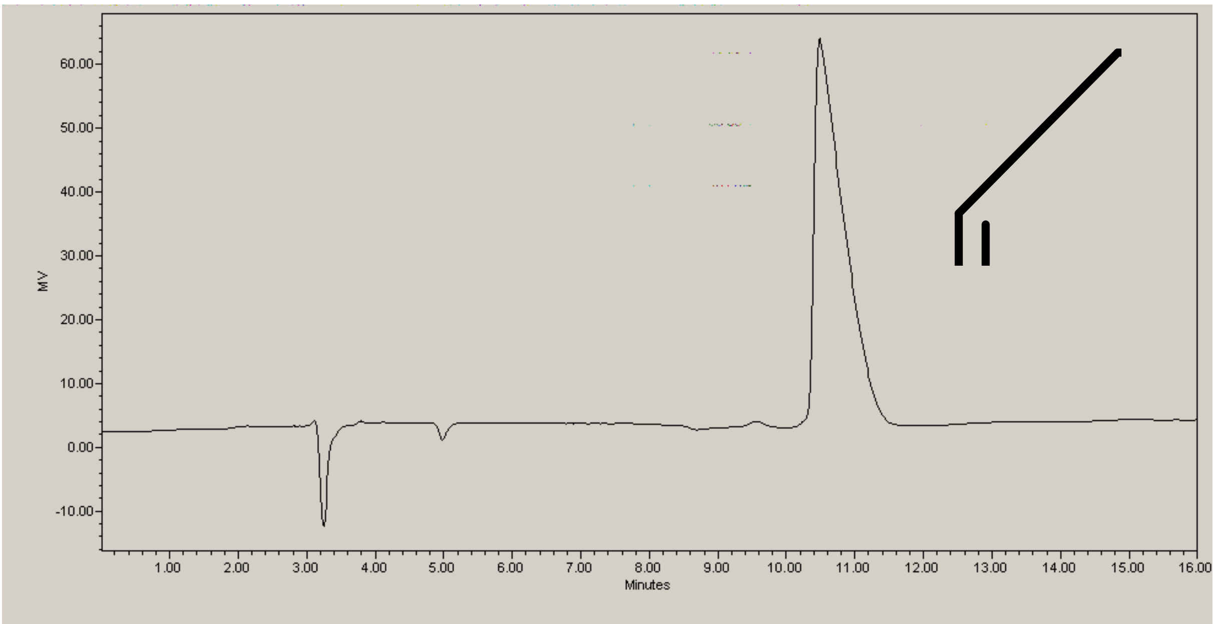

*Diol **2b***

3) NMR Spectra

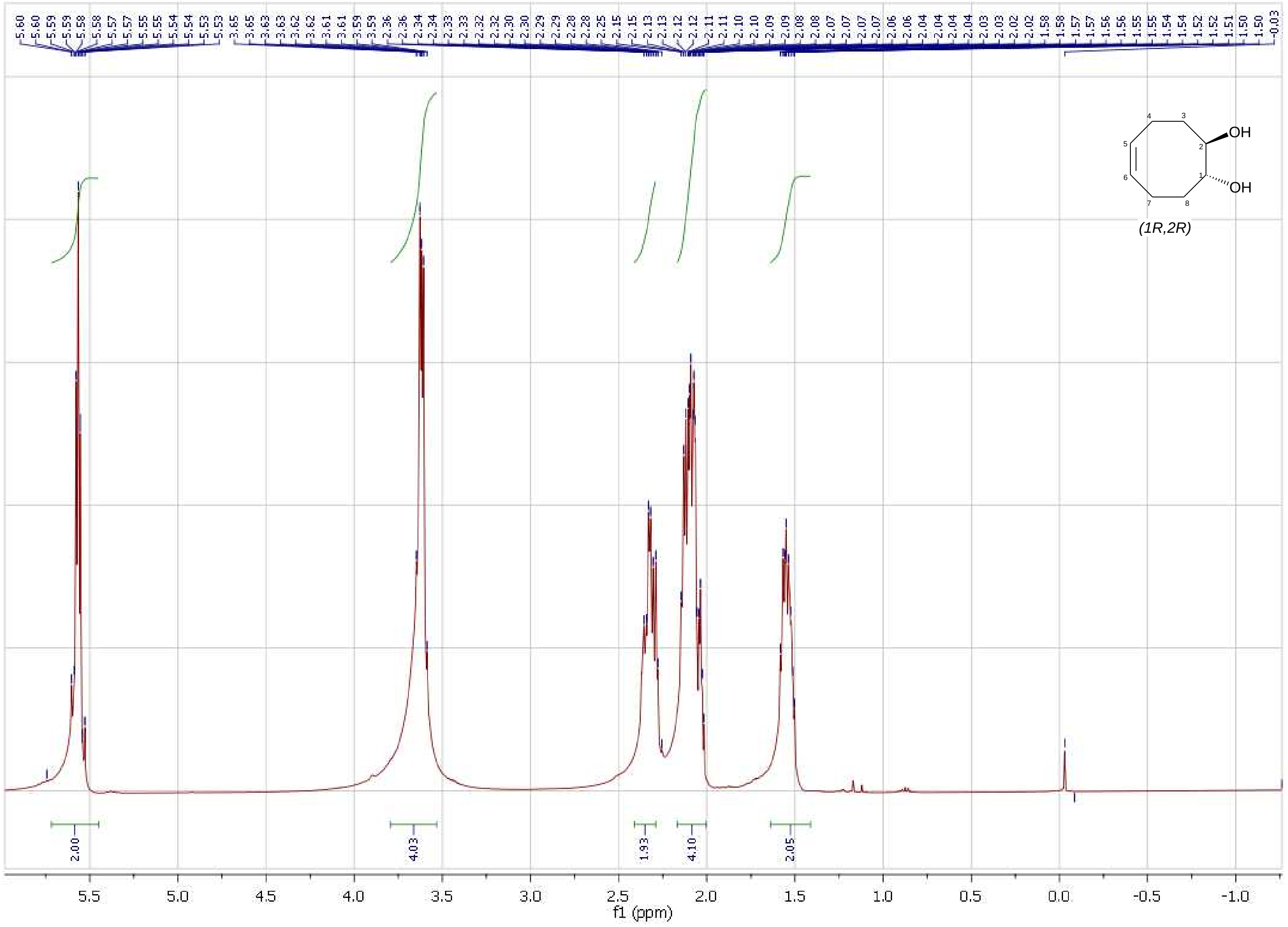

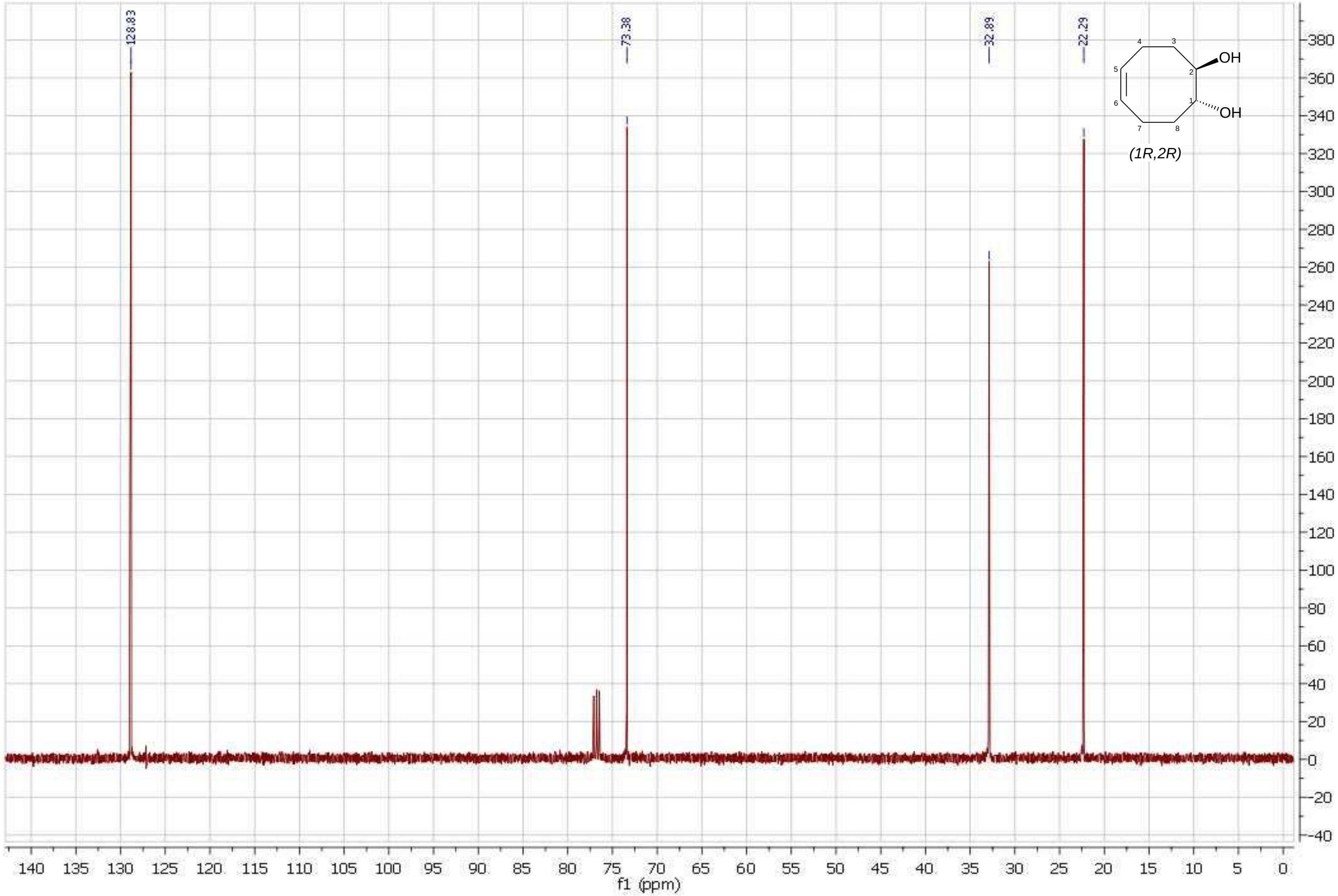
128.83
73.38
32.89
22.29
OH
OH
(1R,2R)
f1 (ppm)

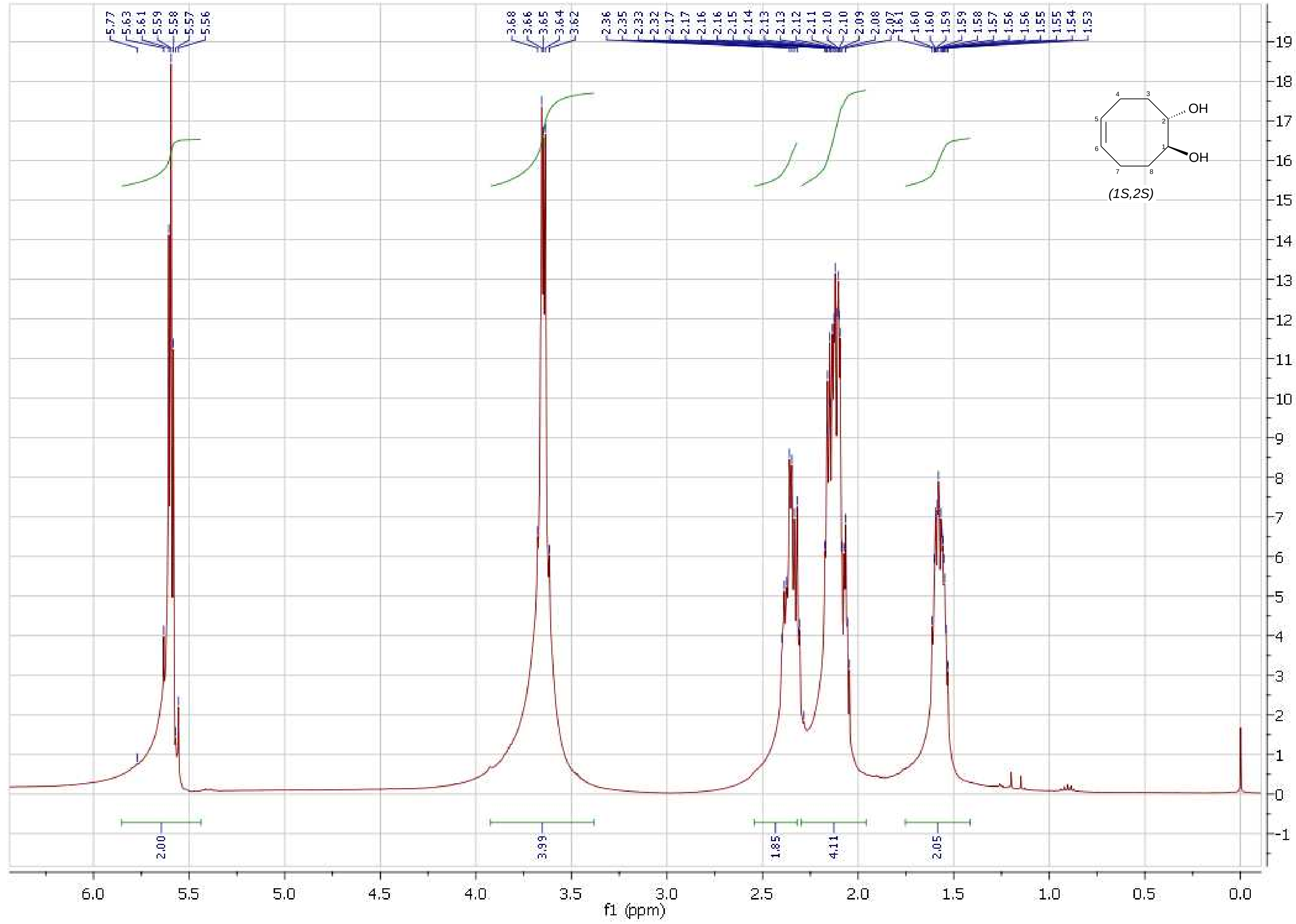
OH
OH
(1S,2S)
f1 (ppm)
2.00
3.99
1.85
4.11
2.05

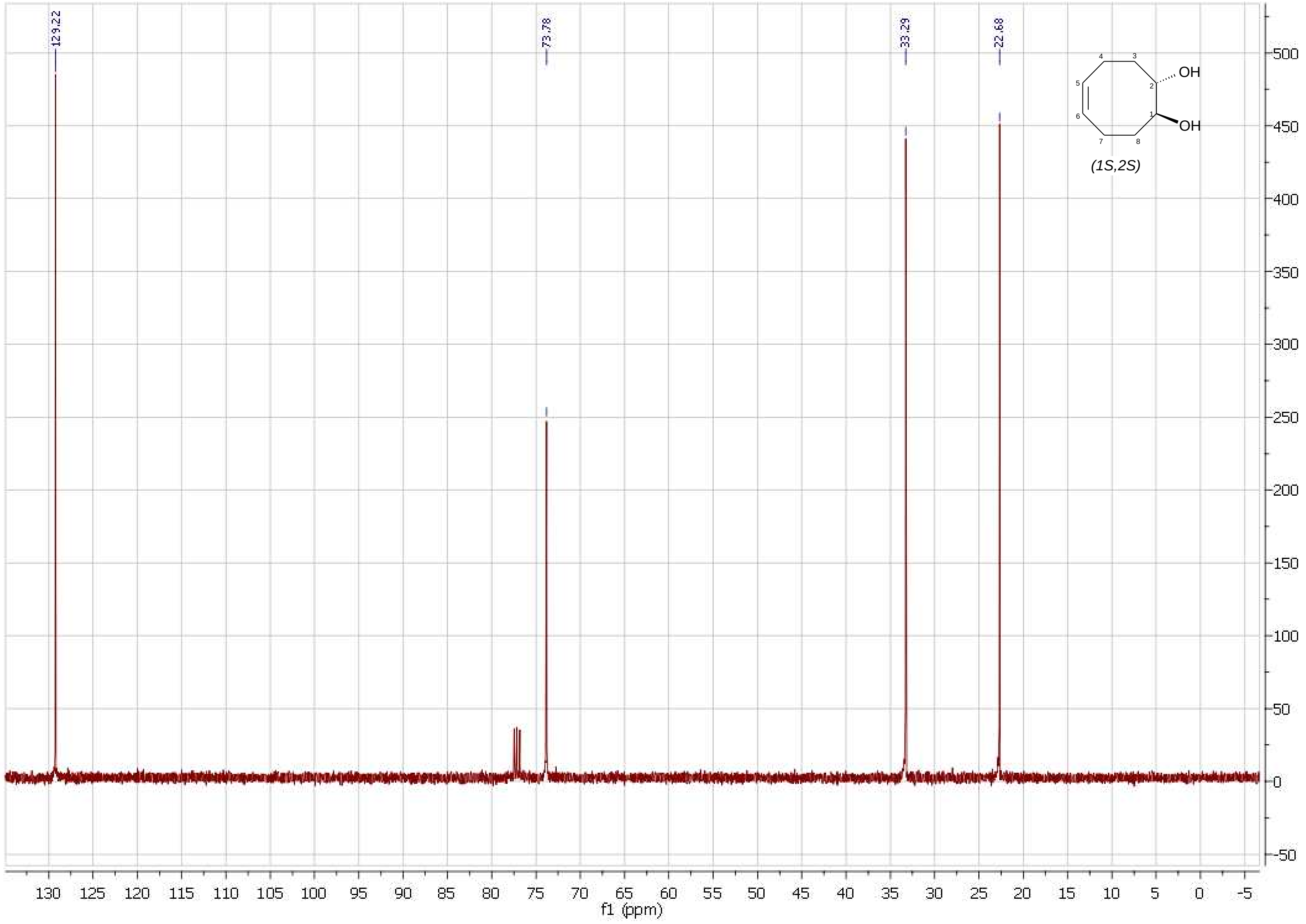

129.22
73.78
33.29
22.68
OH
OH
(1S,2S)
f1 (ppm)

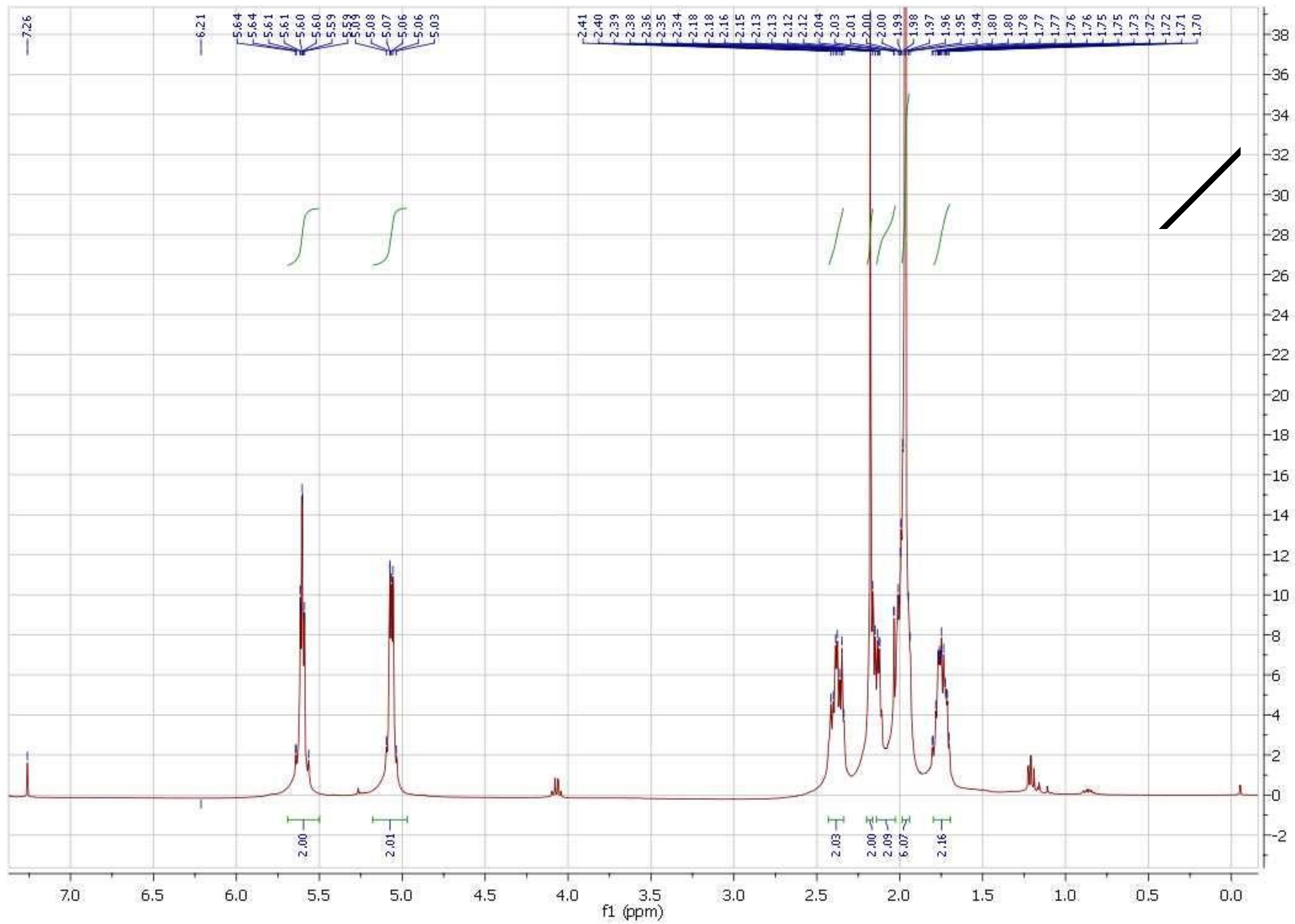
f1 (ppm)

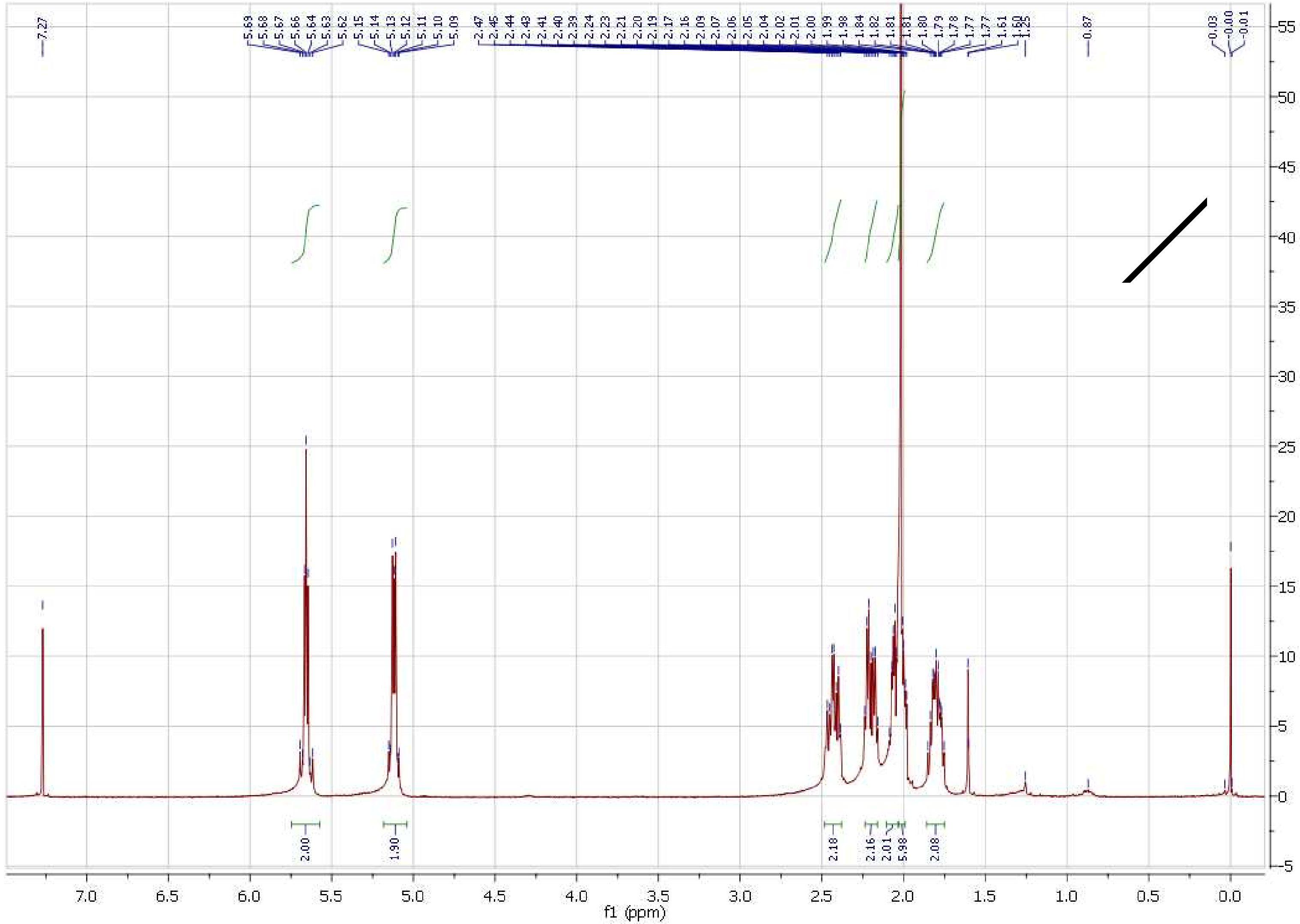
f1 (ppm)

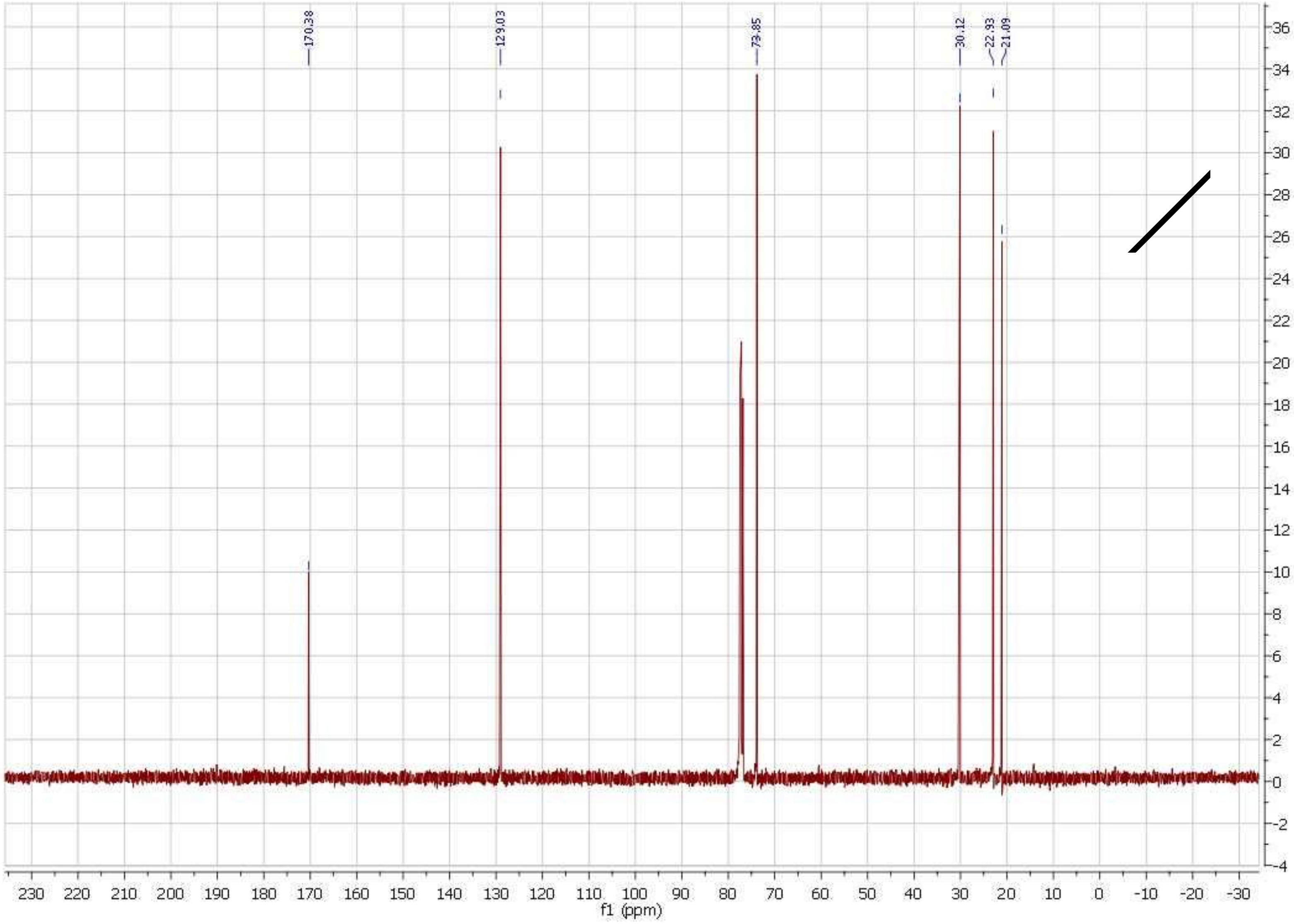
170.38
129.03
73.85
30.12
22.93
21.09
36
34
32
30
28
26
24
22
20
18
16
14
12
10
8
6
4
2
0
-2
-4
230 220 210 200 190 180 170 160 150 140 130 120 110 100 90 80 70 60 50 40 30 20 10 0 -10 -20 -30
f1 (ppm)

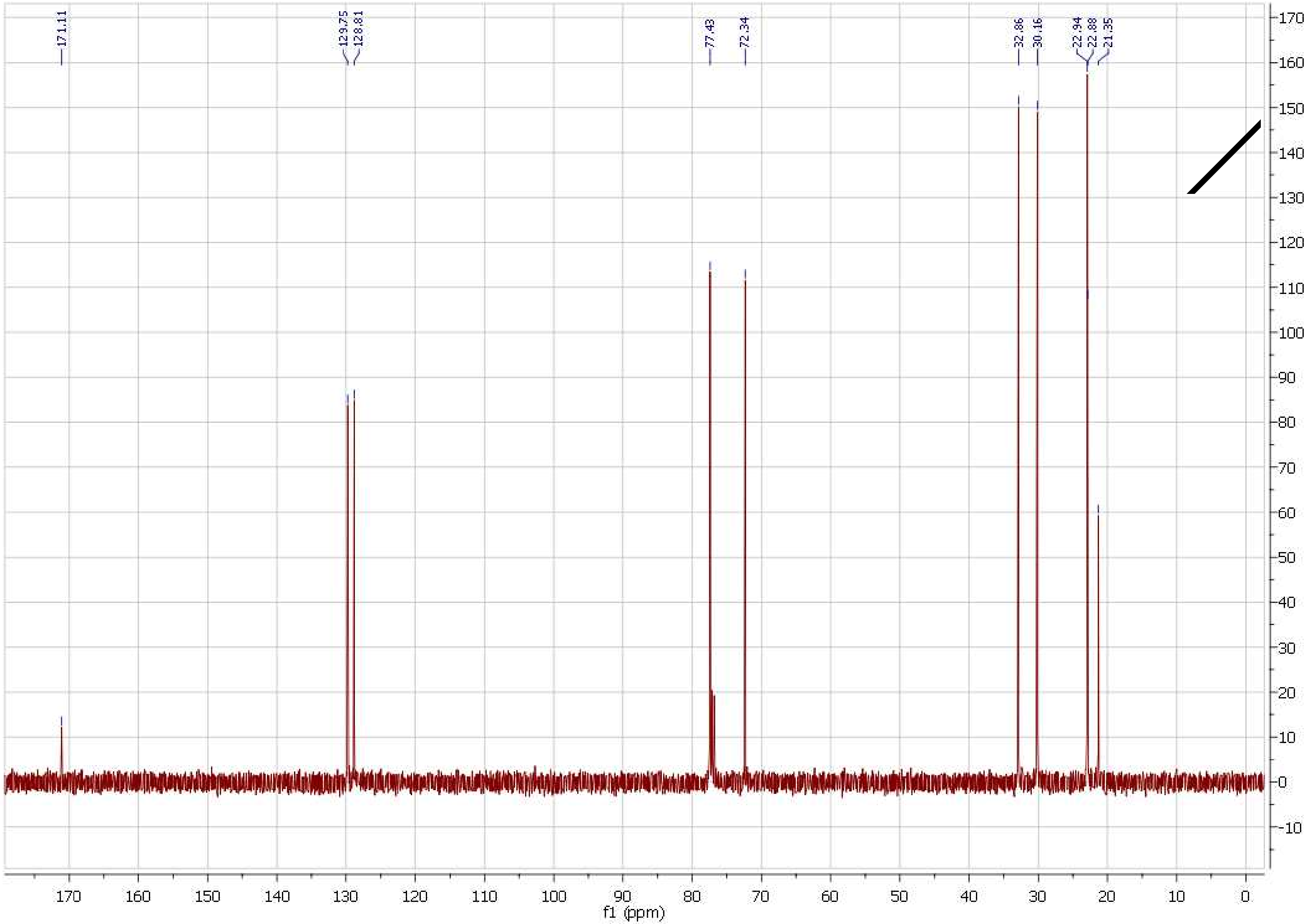
171.11
129.75
128.81
77.43
72.34
32.86
30.16
22.94
22.88
21.35
f1 (ppm)

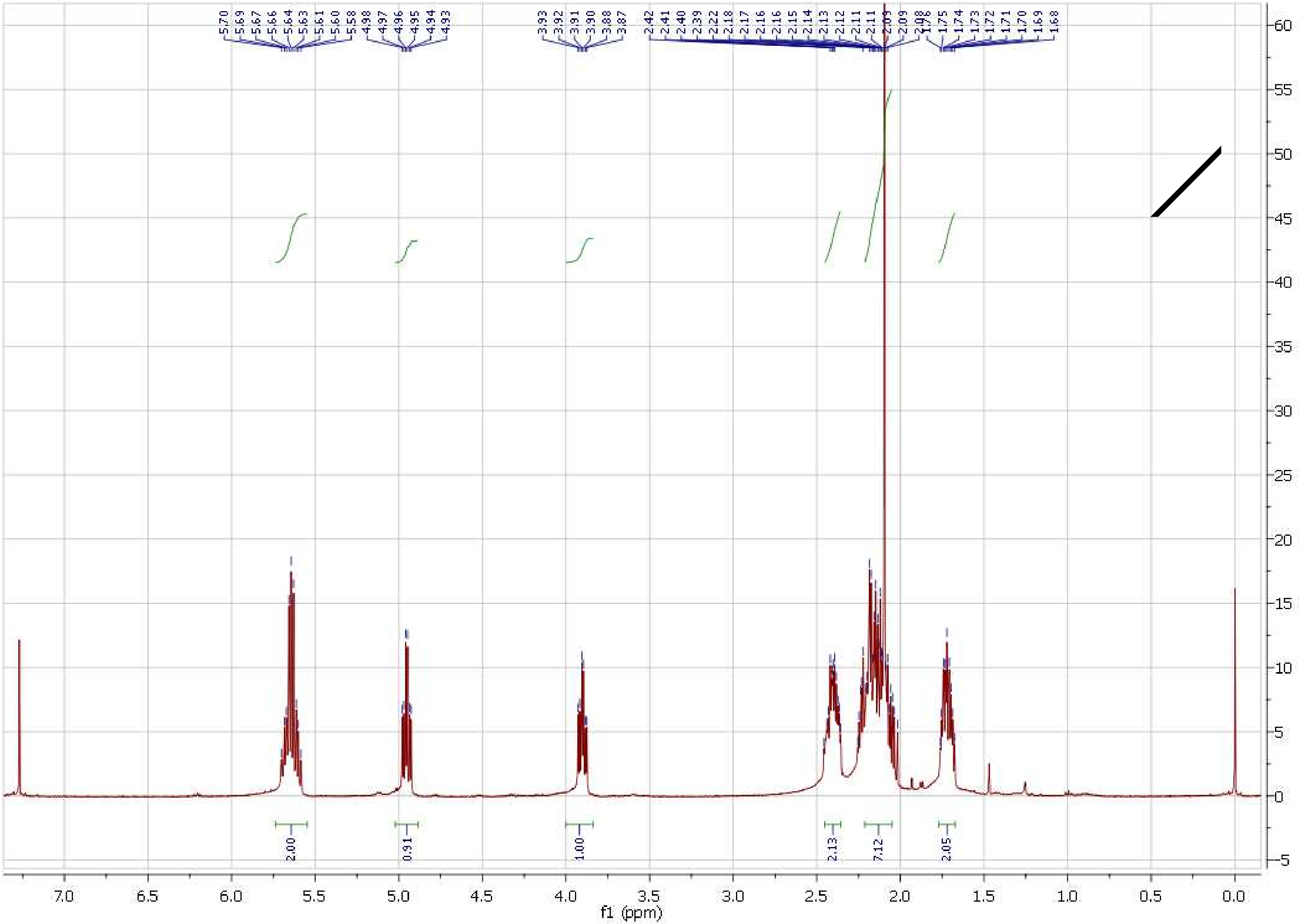

Supplement: Supplementary file 1 [file molecules-19-09215-s001.pdf]
